# The Age Factor in the Analysis of Occupational Risks in the Wood Industry

**DOI:** 10.3390/healthcare10071355

**Published:** 2022-07-21

**Authors:** Noelia Araújo-Vila, Diego R. Toubes, Jose Antonio Fraiz-Brea

**Affiliations:** Business Administration and Tourism School, University of Vigo, 32004 Ourense, Spain; drtoubes@uvigo.es (D.R.T.); jafraiz@uvigo.es (J.A.F.-B.)

**Keywords:** occupational risks, occupational safety and health, age, senior, wood industry

## Abstract

Workers in the wood industry are continually exposed to a wide range of risks. Some risks are potentially high and may lead to serious work-related accidents or occupational diseases. It is a sector where physical work is predominant and where high-risk machinery is used. There is also the age factor, as the age of the workforce increases the risks of loss of skills, particularly physical skills. This study analyses the impact of age on the occupational safety and health management in the wood industry. To this end, a qualitative analysis was carried out through semi-structured in-depth interviews. A total of 52 interviews were conducted with wood-based entrepreneurs, occupational safety technicians and experts from Galicia (Spain). The results show that there is a growing concern to integrate the older group in occupational safety and health management due to the increasing work life. The older group is not the one with the greatest number of occupational accidents (8.3%), but rather the one that needs better working conditions in the face of physical deterioration, considering that a large proportion of senior workers is transferred to administrative tasks. Consequently, the proportion of older workers performing physical tasks, which are the tasks with the highest associated risk, is lower.

## 1. Introduction

Health is understood to be the state of complete physical, mental and social well-being, not just the absence of disease [1,2,3]. Therefore, occupational risks can be defined as those work situations that can disrupt the feeling of well-being and the threefold physical, mental and social balance of the working staff [4,5,6,7].

All short- and medium-term demographic forecasts point to an ageing European population, with a marked predominance of older workers in the labor market. This ageing poses a challenge for companies in the management of their workforce, which means that they have to adopt and develop specific measures to protect and prevent occupational risks that are adapted to their conditions. In addition, company managers must ensure that the performance of work does not contribute to an accelerated deterioration of workers’ health, with the attendant risks and costs. With a suitable working environment, the aim is to maintain the skills and abilities of these workers for longer, and to make possible the objective set by European and Spanish policies of prolonging the working life of these workers [8].

In the face of an ageing population, increasing life expectancy is a major social step forward, but it also has important socio-economic implications, both for businesses and for society at large. Occupational safety and health management for older workers has become a priority as people work longer and the growing proportion of this segment of the population [9].

On the other hand, wood processing involves different types of specialized machinery. These processes require the existence of companies specifically dedicated to this economic activity, often micro-enterprises employing a very small number of people and working without observing basic labor safety standards [10].

In the case of the wood-processing sector (including felling, pruning, transport, quartered, truncation, drying and brushing the wood) workers are exposed to a wide range of risks on a daily basis, some of which are potentially serious and may lead to serious accidents at work or occupational diseases [11,12]. This is a sector in which physical work continues to be the protagonist, in addition to the use of high-risk machinery [13]. There is also the age factor, since the age of the working staff increases risks due to the loss of certain skills, particularly physical skills.

The occupational safety and health management in this sector must be very rigorous. Given that the damage can be very serious, all necessary precautions must be taken [14]. To do so, first of all, workers must know what risks they face and thus know how to prevent them, meaning prevention is the best option [15,16,17].

The wood industry is a sector with a high accident rate. In 2018, in the autonomous community of Galicia (Spain) there were a total of 1120 accidents without sick leave and 227 with sick leave, 4 of them fatal and 11 serious. This is a sector in which the majority of workers are in the 35–44 age group; however, in 2018, 8.3% of accidents occurred in persons over 55 years of age, a minority age group [18]. In other words, although the over-55 age group (which accounts for 18.9% of the total) is not the largest in the sector, it accounts for a significant percentage of accidents. It is striking that although part of this group is relegated to administrative tasks, it still accounts for 8.3% of accidents in the sector. This percentage should be lower because of the very small number of employees over 55 years of age engaged in field work. Companies can prevent work from contributing to premature wear and the onset of work-related illnesses by preventing occupational risks and paying attention to particularly sensitive workers in the light of working conditions and the environment, as well as developing strategies for adapting workplaces. 

Therefore, it is necessary to emphasize the risks associated with the older group as the most vulnerable group, due to the decline in physical abilities at the end of the working life.

Therefore, the following objectives are set out in this paper:

- To analyze whether the age factor is considered in preventive measures in the wood industry.
- To know the main accidents or pathologies of the older group in the wood industry.
- To develop recommendations to improve prevention in the wood industry.

In order to achieve these objectives, first of all, the methodology of the study is explained (qualitative analysis via in-depth interviews) and a review of the main risks occurring in the wood industry is carried out. The main results are then presented and discussed, divided into three blocks according to the three sections in which the interview is structured. Finally, the discussion and main conclusions are presented.

## 2. Theoretical Review

Since the late 1990s, employment growth and longer working lives have been key objectives of national and European policies. The EU-27 employment rate for people aged 55–64 has increased from 40.5% in 2005 to 58.5% in 2018 [19]. Age-induced deterioration primarily affects physical and sensory abilities, which are the most relevant for heavy physical work in the manufacturing sector. Likewise, job changes due to age are more important and frequent in some professional activities than in others. Proper job layout design therefore benefits all age groups, including older workers [19].

This new situation is leading to special attention being paid to the group of senior workers (over 55 years of age). The proper treatment of this group is a major challenge, both at the national and international policy level and for the companies themselves. Among the concerns expressed in relation to this group, the following should be highlighted at a generic level [20]:

- Study of the characteristics of older workers.
- Develop work systems that help this group to exercise their skills effectively.
- Adapt risk prevention and safety at work to the characteristics of this group.
- Develop relevant criteria, based on different factors, to determine the retirement age.
- Flexibility and diversification of the conditions for determining the end of the working life of older workers.
- Adaptation of working conditions to the needs of this group.

In the wood-processing industry, round wood is used to produce a marketable wood product, but not directly consumable, and a second processing would therefore be necessary to produce a final product [21,22,23]. This first phase would include activities such as [24,25]:

- Felling
- Pruning
- Transport
- Quartered
- Truncation
- Drying
- Brushing [26,27].

**Figure 1:**
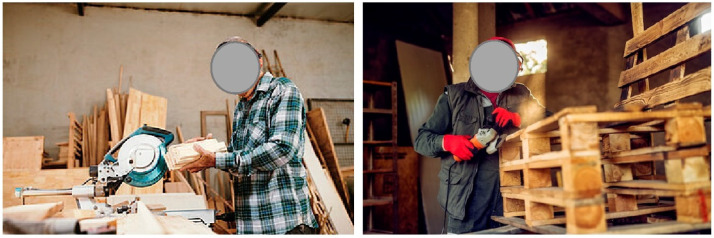
Examples of a senior worker in wood industry.

In the case of wood panel and veneer processing, the wood industry is represented by large companies. The mechanization of operations has been modernized and technologically equipped. The competitiveness of the sector is based on technological efforts aimed at automation and the possibility of creating new products [26] (Figure 1).

The wood processing sector in Galicia is one of the main industries of this community. The Galician Wood Cluster [24] points out that Galicia is the national leader in the sector—50% of the timber cut in Spain comes from the mountains of Galicia—and is the ninth largest forest power in Europe.

In 2017, Galicia recorded a historical value in terms of volume of timber cut, exceeding 8.5 million cubic meters for the first time. Exports exceeded 800 million euros. This represents 47% of the timber cut in Spain, 1.9% of the round wood produced in the EU-28 and 3.5% of the production in the Eurozone. These figures were maintained in 2018 [28].

The timber sector in Galicia is made up of more than 3000 companies that generate wealth, especially in less industrialized environments. In more than 20 counties, this industry ranks among the top three industrial sectors in terms of job creation. The value chain accounts for 12% of industrial employment in Galicia and 1.8% of the community’s GDP. In addition, Galicia has companies in this sector that are benchmarks in design and innovation [29].

The wood industry is also one of the industries with the highest associated risks. The fact that physical work has an important role implies a greater chance of accidents, in addition to other occupational diseases [30]. There is therefore a great need to focus on prevention in the wood sector.

### Risk Factors

Ergonomic risk factors are very common in this industry (wood sector), especially for people over 55 years of age [31]. Over-exertion produces musculoskeletal disorders (MSDs) in workers, e.g., inflammatory or degenerative pain and injuries usually in the back and upper extremities [32,33,34,35,36,37]. Ageing leads to physical deterioration and increases the incidence of these risks, especially in the sector under study where physical exertion is part of the work routine.

Ergonomics [38,39,40,41] is defined, according to the International Ergonomics Association [36], as “the scientific discipline concerned with the understanding of interactions among humans and other elements of a system, and the profession that applies theory, principles, data and methods to design in order to optimize human well-being and overall system performance”. This definition appears in Spanish technical standards such as the UNE EN-614-1: 2006 [42] and UNE-EN ESO 6385: 2004 [43].

To carry out a comprehensive ergonomic assessment [44,45,46,47] that considers all dimensions (task, worker and working conditions), the National Institute for Occupational Safety and Health (NIOSH) proposes a series of guidelines to address this issue: what are the factors to consider and the identification of those that require in-depth analysis [48]?

Ergonomics considers physical, environmental, organizational and psychosocial factors (cognitive and social) [49,50,51,52] with a holistic approach [53,54,55,56]. Among the main factors [57] we can mention four: (i) forced postures, (ii) repetitive movements, (iii) manual handling of loads and (iv) application of forces. The above are risk factors specific to the sector of the first processing of wood, and to these are added others, such as lighting or vibrations (Table 1). As workers age, they become more vulnerable to such risks, leading to more absences from work [8,9].

The main ergonomic risks usually come from adopting forced postures, performing repetitive movements, handling loads and applying forces during the working day [62,63], which is common in the wood sector [64].

Ergonomic risk factors are therefore those working conditions or demands during repetitive work that increase the probability of developing a pathology.

A second factor is psychosocial factors, which are “those conditions which are present in a work situation and which are directly related to the organization, content of the work and performance of the task, and which have the capacity to affect both the well-being or health (physical, mental and social) of the worker and the development of the work” [65]. Examples of these factors are:

- Absence of breaks
- High work rate
- Lack of control over work
- Repetitiveness and monotony
- Lack of information and training
- Shifts and nighttime
- High level of attention during the activity
- Insufficient communication with superiors
- Lack of relationships with colleagues

Scientific evidence underlines the relationship between exposure to psychosocial risk factors at work and the probability of adverse effects on workers’ health (physical, cognitive, emotional and social) and safety [66]. Such exposures increase the probability of the occurrence of situations of tension or violence in any of their manifestations (commonly referred to as “psychosocial risks”) that may have negative effects on people’s safety and health and affect certain occupational health indicators: absenteeism, productivity, satisfaction, turnover, work environment and stress [67,68,69,70,71].

In addition, although workers in the wood processing sector are exposed to occupational risks common to other activities, they are also involved in different processes that expose them to specific risks associated with this activity [72]. These risks include:

- Use of hazardous machinery
- Exposure to high noise levels
- Use of certain substances or inhalation of wood dust [73].

## 3. Methodology

To achieve the objectives of this study, a qualitative research methodology was used through in-depth interviews with experts. This method is characterized by being a technique for obtaining direct information, being flexible (in this study an interview of semi-structured questions is used) and analyzing the information obtained integrating the beliefs, values, desires and attitudes of the interviewees [74]. The in-depth interview is intended to describe the phenomena of interest, as well as human behavior. It is a systematic way of interpreting reality and understanding of phenomena through instruments that provide a vision of reality, opinions and experiences, either of researchers, observers, informants or research participants [75].

### 3.1. Sample

The qualitative technique selected was the in-depth interview. There are 3000 companies dedicated to the processing of wood in Galicia (finite population). We coded in an Excel sheet, and 52 were randomly chosen (with a maximum error of 12% at a confidence level of 95%). In each of them, the expert or person responsible for occupational risks was contacted to be interviewed. We conducted the interviews with this panel of 52 experts chosen for their level of knowledge in the subject and with different approaches, specifically professionals from the wood sector and the field of occupational safety and health in Galicia (Spain). First, we contacted the Association of Entrepreneurs of Primary Processing of Wood, based in the province of Lugo. This association provided the contact in for the sector’s experts in Galicia with whom the interviews were conducted.

The sample is made up of individuals aged between 32 and 60 years, and 68% of them are men. Sixty percent of the individuals in the sample have a high school education and the remaining 40% have studied at a university. In terms of jobs, 40% of those interviewed are occupational risk prevention technicians in the company, another 40% hold managerial positions in the company in occupational risk, and the remaining 20% are occupational safety and health technicians who have been subcontracted by the companies because they do not have their own staff to carry out these tasks.

### 3.2. Data Collection

The interviews were conducted by telephone during the months of July to September 2020. The state of alarm in the study area due to the COVID-19 pandemic forced the closure of a large number of business activities. For this reason, and for security reasons, face-to-face interviews were ruled out and interviews were conducted by telephone. Each of the interviews lasted approximately 50 min and included 10 questions, covering the following aspects (Appendix A):

- SECTION I. Inclusion of the age factor in occupational safety and health (OSH) management. This section consists of six questions that seek to delve into the specific risks at work of people over 55 years old, the current situation regarding age in occupational safety and health, especially in companies in the wood sector.
- SECTION II. Ageing and occupational accidents in the sector. Three questions were asked to provide information on accidents and pathologies in the wood sector by age factor and on the risks associated with people over 55 years of age, particularly ergonomic and psychosocial risks.
- SECTION III. An open-ended question was asked on recommendations to improve the OSH management in the wood sector.

Experts from the area of risk prevention at the University of Vigo and experts from the wood sector in Galicia were consulted for the design of the interview. A preliminary interview was conducted as a pre-test with five of these experts.

### 3.3. Data Analysis

All interviews were transcribed verbatim for further analysis. A thematic analysis and coding techniques were then carried out in order to identify the main themes in the interview transcripts. Thematic analysis is defined as an accessible and flexible method of qualitative analysis aimed at identifying and analyzing common data and patterns within a set of information [75]. According to this technique, the transcribed information is reviewed by the researchers in order to analyze the content in depth and extract the relevant information [75]. In a first phase, the repeated answers that follow a pattern are identified, being the most relevant of the interview because they are shared by several of the interviewees. There is also a need for a prior review of the issue, in this case the occupational safety and health management. In a final stage, the results derived from the questions raised in the research are published.

## 4. Results

### 4.1. OSH Management and Age Factor

Age is a very relevant factor in this sector as it is heavily dependent on physical work and age causes certain skills to be reduced. Most of the workers in this sector are under 55 years of age, so the largest number of accidents is in the 35–54 age group, accounting for 82.4% of accidents. Despite the fact that people over 55 years of age are in the minority in the sector, the number of accidents reaches 8.3%.

One of the experts thinks that the group of workers over 55 is much less likely to have accidents than younger people. This expert states that “for every 10 accidents there is one involving a person over 55 years old” and “the normal thing for people aged 55 and over are occupational diseases and not occupational accidents, resulting from shoulder problems, elbows, overwork…” In short, “younger people usually have more occupational accidents, and older people have more occupational diseases. In the case of occupational diseases, 90% of cases would be people aged 55 or over”.

The interviewee states that, at a statistical level, there is no higher incidence of age-related occupational accidents: “For example, they [older workers] have fewer reflexes, but they are more careful, and ergonomically they do not suffer more muscular problems than other age groups. We have more problems, such as sprains, in young people than in those over 55”. In addition, the interviewee adds that older workers are used to doing physical work, partly because they are mostly from rural areas and have been working on family farms since their young age. On the contrary, many young people live sedentary lives and may develop more joint and muscular problems in the future than previous generations.

Analysis of the information obtained from the expert interviews, together with the information drawn from the various documentary sources, makes it possible to identify a number of needs in the OSH management when it comes to managing ergonomic and psychosocial risks in relation to age.

The first need identified stems from the lack of consensus among the companies in the sector on how the age factor is being addressed in the OSH management. One of the interviewees states that the age of the workers is taken into account in the performance of the different tasks, for example, the risk at each job is measured by age in order to analyze how it affects the worker’s vision or hearing. Another interviewee repeated the same argument, stating that their company does take age into account: “An example of this could be the measurement of occupational risks for each job”. However, this approach cannot be applied to the whole sector, as one interviewee says, “I don’t know about the rest of the sector. The reality is that there is not much difference, taking into account age, in terms of the number of casualties from work and the number of accidents, but we are aware that there are body capacities that are deteriorating, especially when you reach the age of sixty”.

Many of the interviewees do not consider the age factor in risk prevention. One expert mentions that “we are doing what the legislation on workers requires: training, information, provide personal protective equipment (…) and health monitoring. If these four points are met, prevention is over”. In his opinion, “the only thing that is being considered, which may be related to age—but which is valid for all age ranges—is what is related to healthy living, such as avoiding fatty foods, cholesterol, being overweight, smoking and alcoholic beverages, and avoiding a sedentary lifestyle…”. This expert affirms that “there is nothing regulated by any discipline that indicates that the age of the worker influences”. Another interviewee points out that he does not consider it essential to incorporate age into OSH management, unless “the chainsaw operators are aged. In this case, it wouldn’t be wrong to make an age approach, because they shouldn’t be doing those jobs… What happens is that in the forestry sector we only find a population of over 55 years old, either on machines, trucks, or sawmills… but in the sawmills there is practically no such population”.

Another interviewee considers that labor shortages in the sector force employers to be more open in hiring, i.e., age is not taken into account for each job (“you hire what you have”). Furthermore, the expert points out that the wood sector is made up of small firms that do not have the capacity to organize work in such a way that the job can be adapted to the skills and characteristics of each individual, such as age. “In a company with 3–4 people, it is very difficult or impossible to adapt the job to a younger person. The size of the company makes it difficult to take such measures”. This expert believes that age needs to be integrated into the OSH management but considers that it is tricky due to the small size of the companies. 

A final interviewee points out that age is not being considered in the management of OSH despite the fact that many regulations on risk prevention have been issued. “As the risk prevention system is set up, the competition makes it impossible to organize the evaluation and implementation work correctly”.

### 4.2. Ageing and Occupational Accidents in the Sector

It seems clear that age must be a factor in occupational accidents. Years of working life may provide certain advantages to older workers, such as experience, ability to solve complex problems, knowledge of small tricks, greater patience and serenity, etc. Numerous studies show that older age does not necessarily affect worker performance. However, the main health problems affecting older workers are those related to physical capacity, cognitive functions, sight and hearing. The deterioration of these capacities can lead to fatal accidents in this sector, where the handling of machinery and large pieces of wood is commonplace. For this group, some of the needs that must be taken into account are the following: the simplification of tasks, the more frequent breaks at work and the adaptation of the workplace to the functions of worker.

The experts interviewed point out that it is common for hard work to affect workers over 55 years of age, but the number of accidents and absences in this age group is not higher than in other groups. Indeed, experts detect age-related pathologies, such as blindness, impaired hearing, blood pressure problems or heart ailments. But, again, the data suggest that it is the younger age groups that have the highest number of incidents at work.

On the one hand, experts do not consider that psychosocial risks are the most relevant in this sector. Thus, one of the interviewees notes that “in this particular area I don’t see many psychosocial risks, from the point of view of stress, I don’t see that it’s relevant… nor do I think that workers are burnt out by low wages…” However, it is pointed out that in the wood-processing sector, a distinction should be made between activities at the first stage of the wood-processing chain, such as logging, which present more “tough” tasks, and activities carried out in sawmills. In the first case, it is shown that an older person may be more tired of doing this work, since they are sometimes carried out in adverse weather conditions, requires more physical activity, and therefore may give rise to some dissatisfaction. Another expert emphasizes the issue of physical deterioration, “we know that with age, faculties are lost, which lead to vision and hearing problems, and the manipulation of postures-loads”. Data from medical reports, which are the most realistic figures in the sector, as well as from accident reports, do not show a higher incidence of occupational risks or illnesses among persons over 55 years of age, whereas more accidents and muscular injuries are detected among younger workers.

Ergonomic risks, such as forced postures, repetitive movements and over-exertion, are therefore identified as the most significant risks for workers over 55 years of age in this sector: “Elbows, lumbar problems… when handling weights… anything that has to do with machine maintenance…which requires some effort, with forced postures…may affect older people more, as they lose strength”. Other risks, which are independent of age, are also mentioned, such as vibrations produced by certain machines, so that a worker who has been subjected to such vibrations for many years may be affected by their health. Another interviewee insists on this type of risk, since the work carried out in this sector requires a lot of effort and physical work, both from the work itself and from the weight and difficulty of movement due to the use of PPE (safety boots, anti-cut clothing) or the operation of machinery when they have to work in the forest. The interviewee also considers that the ergonomic risks are very important in relation to age when people work in sawmills, although in this case they also depend on the degree of mechanization and modernity of the plant: “From an ergonomic point of view, the handling of loads, forced postures, etc. should be highlighted when working on the hill. In sawmills, the main risk is the handling of loads, sometimes the result of poor work organization or the lack of mechanical means”.

In summary, the main ergonomic risk factors identified by the interviewees are the following: forced postures, over-effort due to manual handling of loads (weights over 25 kg), and repetitive movements, both in sawmills and due to the use of machinery, in which the same muscle groups are continuously moving. Psychosocial risks are not the most prominent in the timber sector; among them, experts point out that the main risk factor is pressure on the worker due to the pace of work. The pace of work is the forest worker’s Achilles’ heel. In some specific cases, “depending on the boss, contradictory orders could be given, some role conflict, but this is not the most common thing”.

### 4.3. Recommendations for Improving Prevention in the Timber Sector

On the basis of the interviews conducted, a number of recommendations or measures were identified that could improve the situation in the sector. Through risk assessment for each job and taking into account variables such as gender and age, the diagnosis and ability to develop a job is improved. If a health problem is identified, the workload for that person may be reduced or the job position changed. This would be a first recommendation to follow as a prevention.

In the view of the majority of experts, preventive measures at work are not being taken in accordance with age, and even less for a given age range. Including the variable age is the primary recommendation derived from this study. One interviewee considers “fundamental to convince and raise awareness among employers, and even employees, of the need to consider and integrate age into OSH”. From her experience, she does not know of any company in the sector that integrates the age factor into the management of OSH.

Another interviewee points out that nowadays there are no specific regulations that establish criteria for promoting measures and actions that consider the age of the worker: “Since the law does not support you, you have nothing to base yourself on when it comes to establishing preventive measures for this age group. We do not have a legal argument linking people’s age to risk prevention that we can use on the basis of the law. In the legislation, no one ever bothered to study age groups, we do not distinguish or treat people differently on the basis of age, and in this sense it is the first legislation that fails. If we don’t have those rules we can’t enforce them”. All this leads to the need to integrate age into the OSH of the sector.

Regarding the specific guidelines to promote the integration of the age factor into the OSH of companies, one interviewee considers both training in manual handling of loads and performing muscle warm-up exercises before starting the day as very important. This expert notes that: “People over 55 make a mistake, they don’t know how to handle loads manually”.

On the other hand, it is important to update the machinery in bad condition. This is a sector that is very dependent on working equipment, and this must be in perfect condition. As one interviewee states, “all companies that are upgrading machinery indirectly have a positive impact on ergonomics, vibrations, noise and the age factor, since the comfort of a new machine is not that of an old machine…even if they don’t realize the advantages it brings”.

On a practical level, the interviewees acknowledge that there is a greater medical monitoring, as more frequent check-ups or specific tests to control age-specific ailments, such as vision, strength, blood pressure, heart disease, etc. An additional measure could be to offer a diet appropriate to the worker’s age.

Another aspect mentioned in this study is the importance of organizing work, as long as the size of the firm allows: “the workplace needs to be adapted for older workers so that they can do the jobs for which they are most qualified or stop doing the jobs for which they have some kind of limitation”.

The term “consciousness-raising” was repeated by several interviewees, both by employees and employers. The interviewees consider it necessary to integrate the age factor into OSH since, in their experience, companies in the sector do not do so. 

Finally, some interviewees advocate the inclusion of new technologies in the sector for safety and accident prevention purposes: “Drones and new communication technologies could be introduced to carry out occupational assessment and prevention”. And they support the creation of heterogeneous work teams, made up of old and new workers, so that there is formative feedback. This training would be beneficial for both older workers and other age groups because it could alert people to the most common dangers and propose appropriate preventive measures. In the case of the senior group, the measures proposed should be oriented toward taking breaks and avoiding effort, and older workers can share their sector experience and know-how with younger workers, thus preventing new workers from making the same mistakes that they have made.

## 5. Discussion

This study shows a significant reality, common to other sectors in our socio-economic environment: the exclusion of the age variable when considering OSH measures. Some of the experts interviewed do not observe a higher accident rate in the wood processing sector among people over 55 years old. However, statistics show that 8.3% of accidents in this sector in Galicia occur in people over 55 years old, and this despite the fact that the most dangerous jobs are concentrated in the younger age range, and people over 55 years old have more weight in other jobs, such as management [29]. Even so, there is a significant percentage of machine operators or lorry drivers over 55 years of age. These jobs entail associated risks due to the deterioration of the abilities of workers of this age or due to their lesser capacity to react to unforeseen events.

The people interviewed in this study do not observe a higher number of accidents among older people than among young people, probably because of their greater experience and caution in performing tasks. There has also been a growing concern within the OSH to give specific treatment to the group of workers over 55 years of age. This concern is mainly based on the deterioration of age-related faculties and the ageing of the workforce in companies. Nearly 19% of the workforce in the sector are over 55 years old. The retirement age is being raised and working lives are being lengthened, so that workers face longer periods of exposure to occupational risks. It is essential to integrate the broader and holistic concept of “active ageing”.

In all jobs and age groups, the aim of preventing occupational risks is to minimize the number of accidents and even eliminate them. As workers age, they lose their physical and mental faculties; this trend must be taken into account in preventive measures so that special treatment is given to older groups of workers. In addition to the usual measures, applied to any age group, other measures should be implemented, such as increased job rotation, shorter work shifts, less heavy work assignments, etc. The group of older workers is of particular interest in today’s society. Given the increase in life expectancy, workers are reaching older ages and must be given special treatment in order to minimize accidents. The natural process of ageing causes a decrease in functional capacities, mainly physical and sensory, which have a direct impact on ergonomic and psychosocial risks.

## 6. Conclusions

At young ages, the exertions do not overly affect the working staff, but over the years it is common to see pains in the joints, muscles and tendons. At that point, casualties increase, resulting in more of these pathologies than of the accidents at work themselves. On the other hand, older workers provide work experience, in the form of technical knowledge and problem solving, a broader and more relaxed view of the problems and, in some cases, more preparation, including physical preparation, for the job than younger workers.

Employees in the age range over 55 tend to be more highly qualified and valued by companies. Specific measures need to be taken for this segment of workers in relation to OSH, such as adapting certain jobs to avoid great effort, providing more rest and specific protections. Otto and Scholl [76] pointed out in their study the need for greater job rotations in order to minimize ergonomic risks in this case.

According to the interviews, we concluded that the over-55 age group does not seem to present more problems than other age groups in terms of work leave or accident reports. Intermediate age groups, with 5 to 10 years of experience, have the highest rate of occupational accidents, mainly due to overconfidence. In reviewing the data on injuries and incidences in the wood sector in Galicia, attention is drawn to the high number of twist injuries among young workers. These sprains occur mainly in industrial buildings rather than on the forest, so it may have to do with sedentary lifestyle and ergonomic postures.

Another measure to consider in order to improve the working conditions of employees over 55 years in the sector is the checking and updating of machinery. All the companies interviewed were updating their machinery, which has a positive impact on ergonomics and lower vibration and noise levels, with positive implications for older workers.

One of the main limitations of this study is the focus of the analysis on a single geographical area, since the conclusions obtained in this research may not be directly extrapolated to other territories with different socio-demographic characteristics or sector structure. However, the high production volume of the wood sector in the geographical area where this study was carried out—one of the most prominent in Spain—makes it suitable for this research. Future studies should analyze other territories and countries with significant figures in the timber sector, thus enabling them to check the results and even make comparisons.

## Figures and Tables

**Table 1:** Ergonomic factors.

Working Conditions/Ergonomic Factor	Components to Be Analyzed
**Physical or Biomechanical Factors** [58]	
Posture/repetitiveness	Posture of the different body groups, maintained posture time, actions performed, levels or degrees of repetition of movements, recovery time, application of force, vibrating tools and impact forces, use of gloves, use of the hand as a tool, thermo-hygrometric conditions, organizational and psychosocial factors, individual factors. In this case, the most common risk factors are the frequency of movements, the postures of the trunk, neck, and upper and lower extremities. These risk factors are applicable both to the more physical jobs in the sector and to administrative tasks (e.g., bad postures resulting from continuous use of computers during working hours).
Manual handling of loads/application of force	Load weight, load position relative to the body, horizontal distance, vertical load displacement, twist and tilt of the trunk, load grip, frequency of handling, load transport, push and pull force, load size, load stability, recovery time, etc. Manual handling of loads is very common in the wood processing industry, depending on the risk factor of the type of lifting: loads, transport, pushing or dragging. Lifting: depends on the weight to be lifted, frequency of lifting, load grip, asymmetry or torsion of the trunk, distance of the load from the body, vertical displacement of the load or duration of the task. Transport: load weight, distance, frequency and accumulated mass transported. Push and drag: force or object and its characteristics, grip height, travel distance, frequency, duration and posture. The physical workload is another aspect of great importance to consider when distributing and organizing the work. It is determined by the physical abilities of the worker, his or her complexion and ability to perform certain actions. Older workers need longer recovery times from physical exertion. Incorrect or inadequate rest may affect the performance of their tasks in the future and cause adverse effects on their health.
**Environmental Conditions** [59]	
Lighting	Illumination level, luminance, luminance and illuminance balance, glare, color temperatures, etc.
Vibrations	Acceleration, frequency, wave direction and type of exposure (whole body, hand-arm).
Thermo-hygrometric conditions	Air temperature, radiant temperature, relative humidity, air speed, etc.
Noise	Sound pressure level, frequency, time variation, information content, etc.
Quality of the indoor environment	Air renewal [60], ventilation, carbon monoxide, carbon dioxide, volatile organic compounds, legionella, etc.
**Individual Factors** [61]	
Gender, age, seniority of position, associated pathologies, lifestyle, level of education, etc.	

Source. Adaptation from NIOSH [49].

## Appendix A. Interview Questions (Translated from the Galician Language)

1. In the area of occupational risk, various international and national regulations stress the importance of promoting preventive measures and actions taking into account the age of workers. In your view, what are the reasons for integrating age into the prevention of occupational risks?
2. What specific occupational risks do you consider exist for working people aged 55 or over?
3. Do you consider that occupational safety and health (OSH) in the workplace is currently being tackled effectively in an age-sensitive manner? If so, how? If not, why do you think it is not being addressed properly?
4. In the specific context of the Galician companies involved in the first processing of wood, to what extent do you consider it necessary to incorporate the age of the worker into OSH management? Please reason your answer.
5. Studies show that in the workplace there are differences in the incidence of accidents, diseases and occupational pathologies due to the age of the worker. Based on your experience and/or knowledge in OSH management in the sector, do you appreciate differences in the incidence of work-related accidents or occupational pathologies in the 1st Wood Processing sector caused by age? and if so, what typology?
6. Could you identify the main ergonomic and psychosocial risks to which workers aged 55 or over in the sector would be exposed and the risk factors that cause them? Considering the age factor, what variables should be taken into account in the OSH management?
7. The Prevention Plan provides guidelines on how to address OSH in the company, analyzing and evaluating the risks and setting out the measures to be implemented. In this respect, and bearing in mind the age of workers, what aspects should be taken into account when carrying out occupational risk assessments in general, and ergonomic and psychosocial risks in particular?
8. What preventive measures are usually taken by companies in the sector, based on the consideration of the age factor?
9. Finally, could you give us specific guidelines to promote the integration of the age factor into the OSH management? Could you provide some examples of good practice on OSH management from an age perspective?
10. Are there any other comments you would like to make on this subject?
